# Parametric characteristics analysis of three cells in 3D and five-directional annular braided composites

**DOI:** 10.1371/journal.pone.0254691

**Published:** 2021-08-04

**Authors:** Weiliang Zhang, Xupeng Wang, Xiaomin Ji, Xinyao Tang, Fengfeng Liu, Shuwei Liu

**Affiliations:** 1 School of Mechanical and Precision Instrument Engineering, Xi’an University of Technology, Xi’an, China; 2 Institute of Mechanical Engineering, Baoji Univ. Arts & Sci., Baoji, China; 3 Department of Industrial Design, Xi’an University of Technology, Xi’an, China; University of Genova, ITALY

## Abstract

On the basis of analyzing the movement law of 3D circular braided yarn, the three-cell model of 3D five-direction circular braiding composite material is established. By analyzing the node position relationship in various cell models, the calculation formulas of braiding angle, cell volume, fiber volume and fiber volume content in various cell models are obtained. It is found that there are four different braiding angles in four internal cells, and the braiding angles in internal cells gradually increase from inside to outside. The braiding angles of upper and lower surface cells are approximately equal. With the increase of the length of the knuckles, the braiding angles of each cell decrease, and the braiding angles of the four inner cells decrease greatly, while the braiding angles of upper and lower surfaces decrease slightly. The results of parametric analysis showed that with the increase of the length of the knuckles and the inner diameter of cells, the mass of cells increased proportionally, while the total fiber volume content of cells decreased. With the increase of braiding yarn number and axial yarn number, the unit cell mass decreases in direct proportion, and the unit cell total fiber volume content increases. Through the research results of this paper, the geometrical characteristics of the cell model under different braided parameters can be obtained, which greatly improves the analysis efficiency.

## 1. Introduction

The 3D braided composites are made by braiding fibers into preforms with specific structural shapes according to certain movement rules, and then compounding, compacting and curing the preforms with liquid matrix. Because the braided fiber as reinforcement has a complex interlaced network structure in space, the 3D braided composite material has many advantages, such as high specific strength and stiffness, excellent impact damage resistance, fatigue resistance and interlayer connection strength. At present, the 3D braided composite material has been widely used in aviation, aerospace, transportation, medical equipment and other fields [1–4].

Because of the good application prospect of 3D braided composites in engineering, the improvement of process structure and the prediction of mechanical properties of 3D braided composites have been widely studied. Lu et al., taking into account the extrusion factors between fiber bundles in the manufacturing process of 3D four-directional braided composites, established the finite element model of 3D four-directional braided composites unit cell by using CAD software, derived the geometric relationship between the braiding parameters and the structural model parameters based on the unit cell model, and calculated the influence of fiber volume content in the cell on the geometric characteristics [5–7]. Zhang et al. established three different solid structure models of inner, surface and corner elements of 3D rectangular braided composites, simulated the mechanical properties of 3D rectangular braided composites with finite element method, and gave the deformation and stress distribution of the three element models, and studied in detail the influence of braiding angle and fiber volume content on the elastic constant of 3D braided composites [8, 9]. Fang et al. chose representative volume element (RVC) to study the compressive mechanical properties of 3D braided composites at different braiding angles. The results show that the compressive mechanical behavior of braided composites with smaller braiding angles is sensitive to the initial defects of braided yarns, and the strength of braided composites with different braiding angles is controlled by different microscopic failure modes. These studies have laid a foundation for the application of braided composites in aerospace and other fields [10].

Many of the above-mentioned literatures have studied the 3D four-way rectangular braided composites, but few have studied the 3D four-way circular braided composites. Lu and Li have studied the parameterization of 3D and four-way circular braided composites, but they have not done in-depth analysis and research on the parameterization of yarn position under different braiding parameters, and different braiding parameters affect the change of yarn position, which is the root of the change of yarn physical and mechanical properties [5–12].

The paper has been divided into five sections. In Sec.2, the movement law of 3D and five-directional circular braided yarn and the forming principle of preform are analyzed. In Sec.3, the coordinates of each node in the cell are calculated by coordinate transformation, and the parametric modeling of the 3D circular braided material cell is completed. In Sec.4, according to the established parametric relationship of 3D circular braided material cells, the influences of input parameters such as the knuckle length and cell inner diameter on cell mass, fiber volume content and braiding angle are discussed. In Sec.5, we state the main conclusions. Through the parametric relationship of three cells of 3D and five-directional annular braided composites established in this paper, the parameters such as cell mass, fiber volume content and braiding angle can be quickly predicted, which provides convenience for the prediction of mechanical properties and parametric modeling of 3D annular braided composites.

## 2. Analysis of the movement law of 3D circular braided yarn

**Fig 1:**
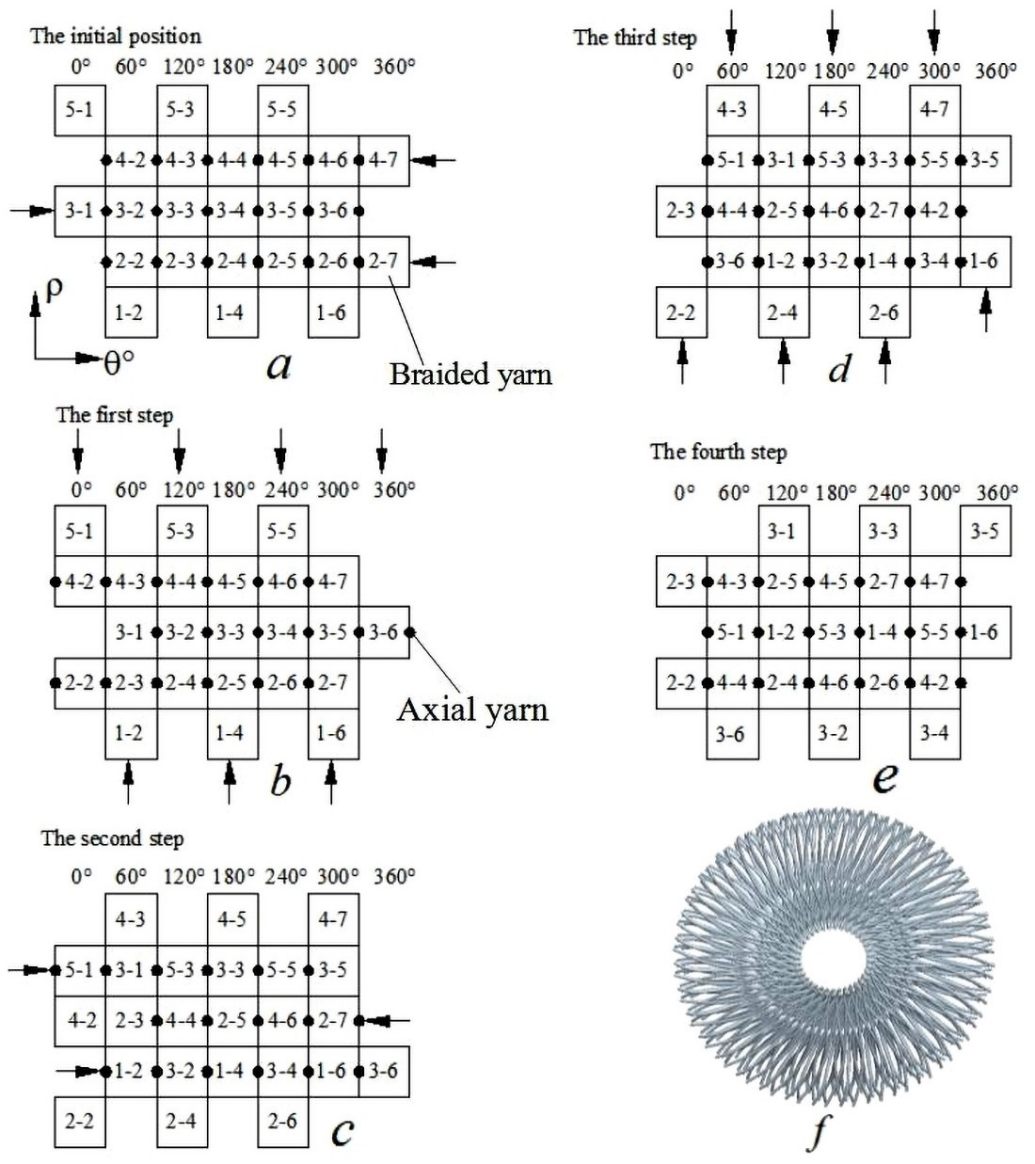
Principle of 3D circular braiding.

As shown in Fig 1, the braided method used in 3D circular braiding is four-step circular braiding. The circular and rectangular braiding methods are similar. The difference lies in that the coordinate system of circular braiding is polar coordinates. The working principle of 3D five-directional four-step circular braiding is illustrated by taking 5×6 (5 layers in Z direction, 6 braiding yarns in radial direction, M = 6, and 6 axial yarns in radial direction, N = 6) as an example.

In Fig 1, a is the initial position, in the first step, on the basis of Fig 1(a), the line carriers move each other, with even lines (lines 2 and 4 in Fig 1(a)) moving in the −*θ* direction and odd lines (line 3 in Fig 1(a)) moving in the +*θ* direction, the result after moving is shown in Fig 1(b). In the second step, on the basis of Fig 1(b), the yarn carriers move mutually, with even columns moving in the direction of +*ρ* and odd columns moving in the direction of −*ρ*, the result after moving is shown in Fig 1(c). In the third step, on the basis of Fig 1(c), the line carriers move mutually, with even lines (lines 2 and 4 in Fig 1(c)) moving in the +*θ* direction and odd lines (line 3 in Fig 1(c)) moving in the −*θ* direction, the result after moving is shown in Fig 1(d). The fourth step, on the basis of Fig 1(d), the yarn carriers move mutually, the even columns move in the −*ρ* direction, and the odd columns move in the +*ρ* direction, the result after moving is shown in Fig 1(e).

In Fig 1, ● refers to the axial yarn carrier, which only moves in the *θ* direction, not in the *ρ* direction. The yarn carrier continuously circulates the above-mentioned four-step movement and gradually weaves into the required preform, as shown in Fig 1(f).

**Fig 2:**
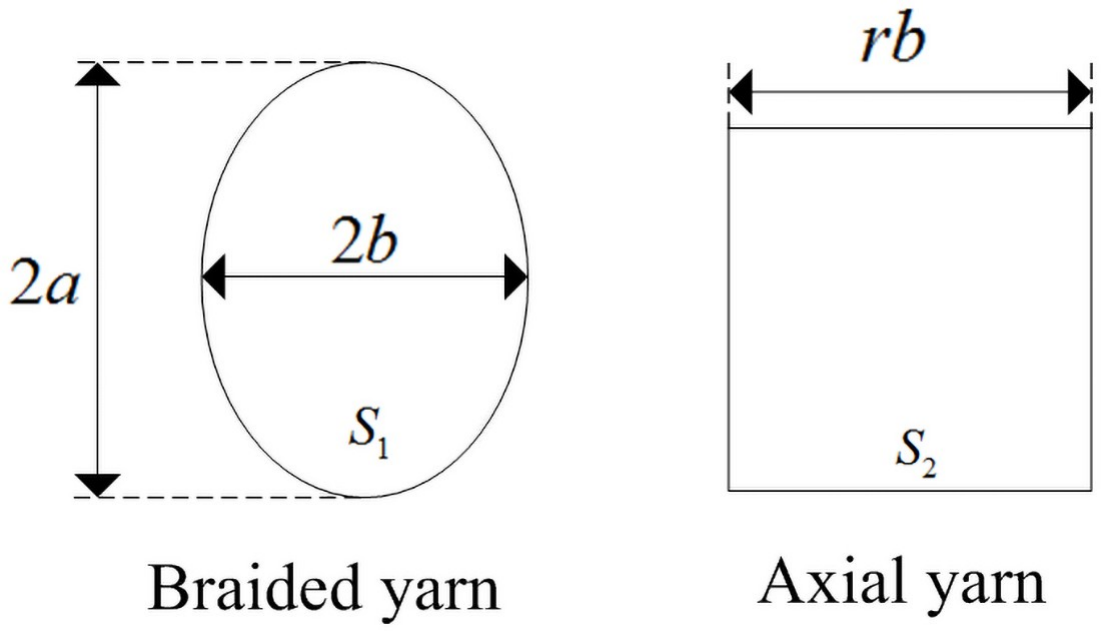
Cross-sectional shape of yarn.

Fig 2 shows the cross-sectional shape of 3D circular braided yarn. The cross-sectional shape of braided yarn is elliptical. The half of major axis of ellipse is *a*, the half of the minor axis of ellipse is *b*, *S*_1_ is the cross-sectional area of ellipse, *S*_1_ = *πab*. The cross-sectional shape of the axial yarn is square, the side length is *rb*, *S*_2_ is the cross-sectional area of the axial yarn, *S*_2_ = *r*^2^ *b*^2^, *r* is the cross-sectional dimension coefficient of axial yarn, which indicates the dimensional relationship between braided yarn and axial yarn [8, 13].

## 3. Parametric modeling of 3D circular braided material cells

**Fig 3:**
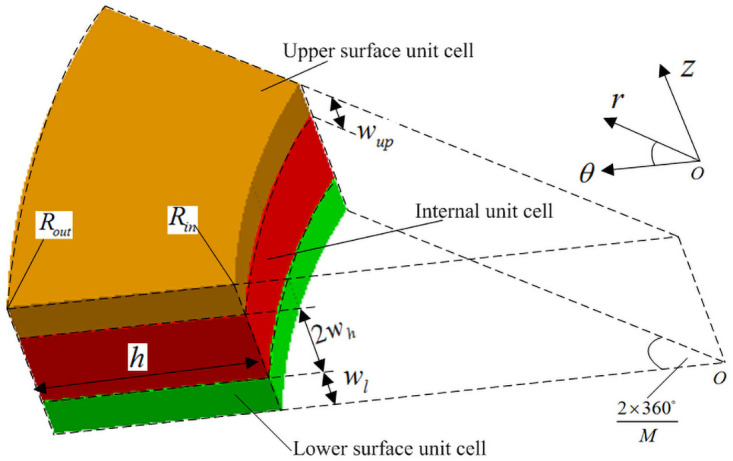
Three cell divisions.

According to the analysis of 3D circular braiding principle, it is found that the braiding yarns in the whole circular braiding preform are 8 kinds of yarns numbered 1–2, 2–4, 2–5, 3–2, 3–3, 4–3, 4–4, 5–3 in Fig 1, which are arrayed in the circumferential direction. Therefore, the microstructure of the annular braided preform should be periodic. In order to improve the modeling efficiency, the minimum research unit of 3D and five-directional annular braided composites is divided into three forms: internal unit cell, upper surface unit cell and lower surface unit cell according to Fig 3, where *w*_up_, *w*_*h*_ and *w*_*l*_ are the height of upper surface, inner surface cell and lower surface unit cell, respectively. Where h is the knuckle length, *R*_*in*_ and *R*_*out*_ are the inner and outer diameter unit cell, respectively [14–18].

### (1) Parametric calculation of internal unit cell

The internal unit cell in Fig 3 can be further subdivided into four sub-regions A, B, C and D with different motion laws, and the sub-regions extend periodically outward along the radial braiding direction.

**Fig 4:**
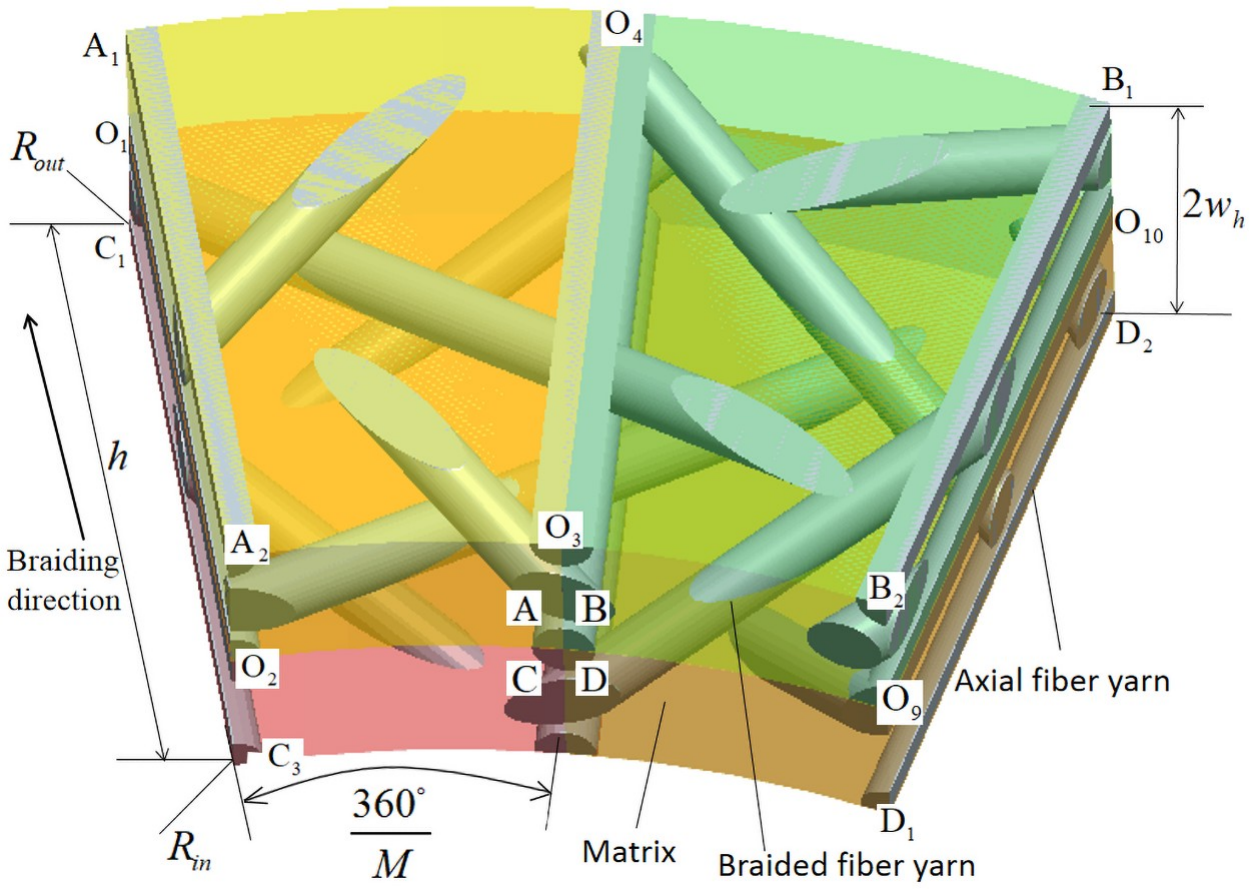
Internal unit cell matrix and fiber yarn.

**Fig 5:**
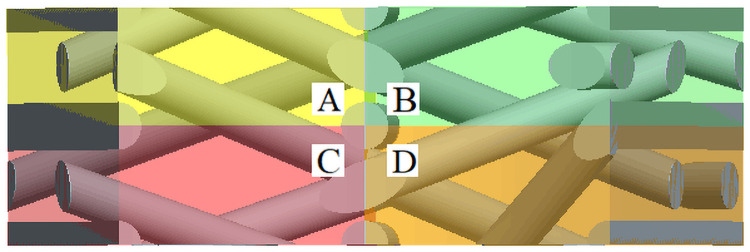
Cross-sectional view of internal unit cell matrix and fiber yarn.

Because there are four steps and one cycle in the braiding process, in Fig 4, the knuckle length h of one node is taken as the width of the inner cell, and the height of inner daughter cell is *w*_*h*_. Fig 5 is a cross-sectional view of four forms of internal single cell matrix and fiber yarn. It can be seen from Fig 5 that the braiding process is actually to interweave yarns according to certain rules.

**Fig 6:**
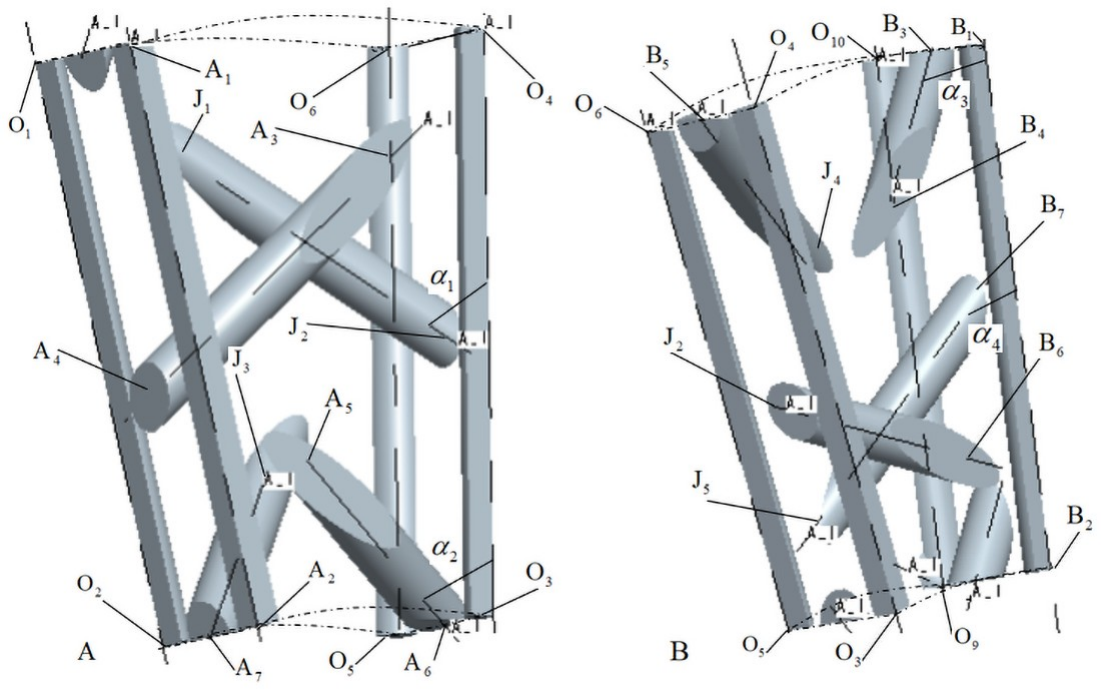
Position relation diagram of internal unit cells A and B.

Fig 6 is a positional relationship diagram of internal cells A and B, defining the angle between internal braiding yarn and braiding direction as internal braiding angle *α*. *α*_1_ and *α*_2_ are internal braiding angles of internal cells A, *α*_3_ and *α*_4_ are internal braiding angles of internal cells B in Fig 6 [19–21].

According to the positional relationship diagram of internal cells A and B in Fig 6, it can be obtained that the parameterized coordinates of A_i_ and B_i_ points in Fig 6 are shown in Table 1 under the cylindrical coordinate system (*ρ*, *θ*, *z*).

**Table 1:** Coordinates of A_i_ and B_i_ points in cylindrical coordinate system.

Position	Coordinate point	Position	Coordinate point
A_1_	$\left(R_{out},\frac{\text{-}360^{{}^{\circ}}}{M},2w_{h}\right)$	B_1_	$\left(R_{\text{out}},\frac{360^{{}^{\circ}}}{M},2w_{h}\right)$
A_2_	$\left(R_{in},\frac{-360^{{}^{\circ}}}{M},2w_{h}\right)$	B_2_	$\left(R_{in},\frac{360^{{}^{\circ}}}{M},2w_{h}\right)$
A_3_	$\left(R_{in}+\frac{3h}{4},\frac{-180^{{}^{\circ}}}{M},2w_{h}\right)$	B_3_	$\left(R_{out},\frac{360^{{}^{\circ}}}{M},\frac{3}{2}w_{h}\right)$
A_4_	$\left(R_{in}+\frac{h}{2},\frac{-360^{{}^{\circ}}}{M},\frac{3w_{h}}{2}\right)$	B_4_	$\left(R_{in}+\frac{3h}{4},\frac{180^{{}^{\circ}}}{M},2w_{h}\right)$
A_5_	$\left(R_{in}+\frac{h}{4},\frac{-180^{{}^{\circ}}}{M},2w_{h}\right)$	B_5_	$\left(R_{out},0^{{}^{\circ}},\frac{3}{2}w_{h}\right)$
A_6_	$\left(R_{in},0^{{}^{\circ}},\frac{3w_{h}}{2}\right)$	B_6_	$\left(R_{in}+\frac{h}{4},\frac{180^{{}^{\circ}}}{M},2w_{h}\right)$
A_7_	$\left(R_{in},\frac{-360^{{}^{\circ}}}{M},\frac{3w_{h}}{2}\right)$	B_7_	$\left(R_{in}+\frac{h}{2},\frac{360^{{}^{\circ}}}{M},\frac{3}{2}w_{h}\right)$

**Fig 7:**
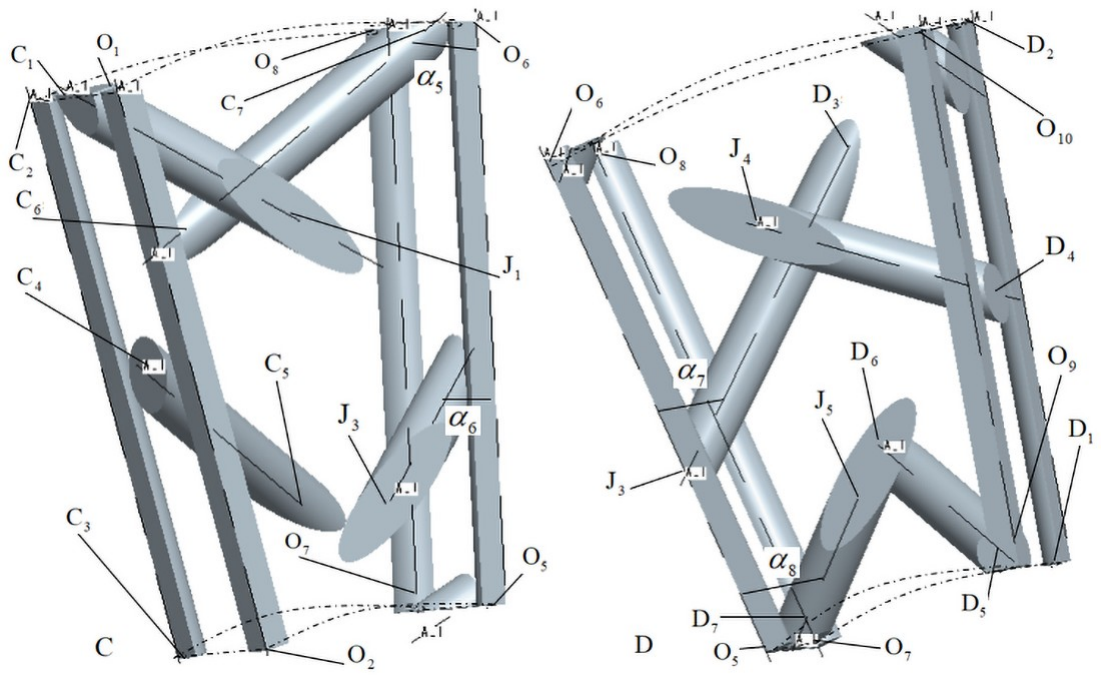
Position relation diagram of internal unit cells C and D.

Fig 7 is a positional relationship diagram of internal cells C and D. *α*_5_ and *α*_6_ are internal braiding angles of internal cells C, *α*_7_ and *α*_8_ are internal braiding angles of internal cells D in Fig 7.

According to the positional relationship diagram of internal cells C and D in Fig 7, it can be obtained that the parameterized coordinates of C_i_ and D_i_ points in Fig 7 are shown in Table 2 under the cylindrical coordinate system (*ρ*, *θ*, *z*).

**Table 2:** Coordinates of C_i_ and D_i_ points in cylindrical coordinate system.

Position	Coordinate point	Position	Coordinate point
C_1_	$\left(R_{out},\frac{-360^{{}^{\circ}}}{M},\frac{w_{h}}{2}\right)$	D_1_	$\left(R_{\text{in}},\frac{360^{{}^{\circ}}}{M},0\right)$
C_2_	$\left(R_{out},\frac{-360^{{}^{\circ}}}{M},0\right)$	D_2_	$\left(R_{out},\frac{360^{{}^{\circ}}}{M},0\right)$
C_3_	$\left(R_{in},\frac{-360^{{}^{\circ}}}{M},0\right)$	D_3_	$\left(R_{in}+\frac{3h}{4},\frac{180^{{}^{\circ}}}{M},0\right)$
C_4_	$\left(R_{in}+\frac{h}{2},\frac{-360^{{}^{\circ}}}{M},\frac{w_{h}}{2}\right)$	D_4_	$\left(R_{in}+\frac{h}{2},\frac{360^{{}^{\circ}}}{M},\frac{w_{h}}{2}\right)$
C_5_	$\left(R_{in}+\frac{h}{4},\frac{-180^{{}^{\circ}}}{M},0\right)$	D_5_	$\left(R_{in},\frac{360^{{}^{\circ}}}{M},\frac{w_{h}}{2}\right)$
C_6_	$\left(R_{in}+\frac{3h}{4},\frac{-180^{{}^{\circ}}}{M},0\right)$	D_6_	$\left(R_{in}+\frac{h}{4},\frac{180^{{}^{\circ}}}{M},w_{h}\right)$
C_7_	$\left(R_{out},0^{{}^{\circ}},\frac{w_{h}}{2}\right)$	D_7_	$\left(R_{in},0^{{}^{\circ}},\frac{w_{h}}{2}\right)$

In Figs 6 and 7, O_i_ is the common node of four sub-regions: A, B, C and D, and the parameterized coordinates of O_i_ in cylindrical coordinate system (*ρ*, *θ*, *z*) are shown in Table 3. In Figs 6 and 7, J_i_ is the connection node of the same yarn in the four sub-regions A, B, C and D.

**Table 3:** Coordinates of O_i_ point in cylindrical coordinate system.

Position	Coordinate point	Position	Coordinate point
O_1_	$\left(R_{out},\frac{-360^{{}^{\circ}}}{M},w_{h}\right)$	O_6_	(*R*_out_, 0°, *w*_*h*_)
O_2_	$\left(R_{in},\frac{-360^{{}^{\circ}}}{M},w_{h}\right)$	O_7_	(*R*_*in*_, 0°, 0)
O_3_	(*R*_*in*_, 0°, 2*w*_*h*_)	O_8_	(*R*_*out*_, 0°, 0)
O_4_	(*R*_*out*_, 0°, 2*w*_*h*_)	O_9_	$\left(R_{in},\frac{360^{{}^{\circ}}}{M},w_{h}\right)$
O_5_	*R*_*in*_, 0°, *w*_*h*_)	O_10_	$\left(R_{out},\frac{360^{{}^{\circ}}}{M},w_{h}\right)$

**Fig 8:**
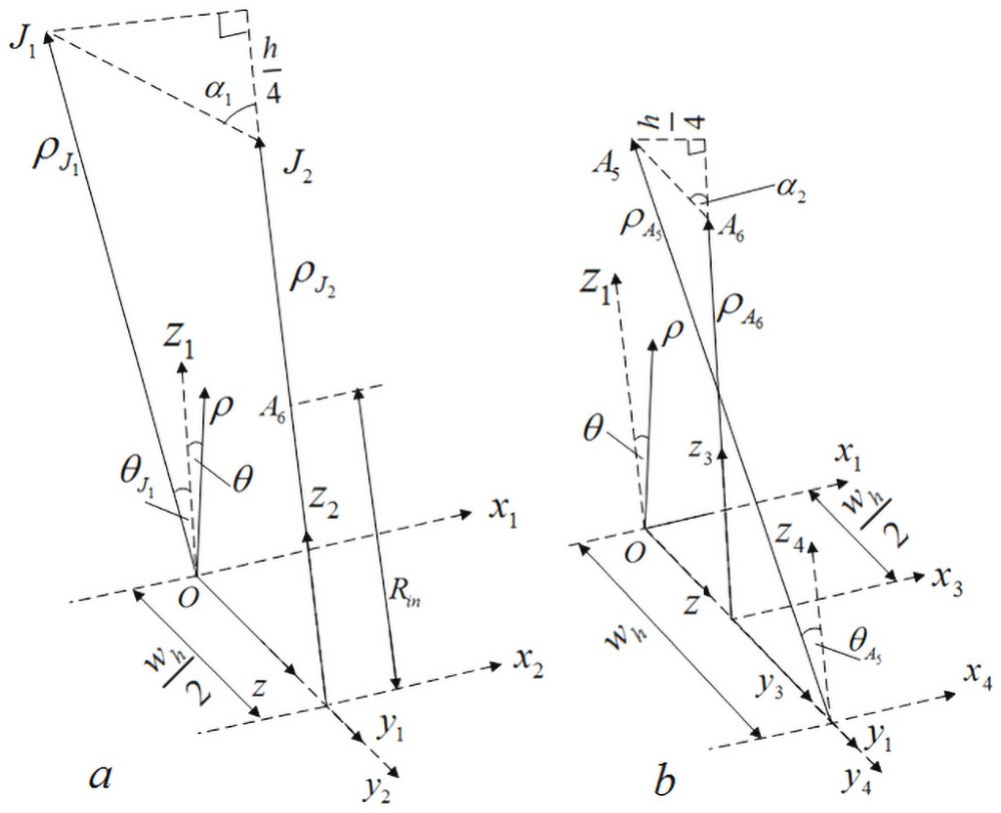
Calculation of braiding angle of internal cell A.

In Fig 8, *ρ* − *θ* − *z* is a cylindrical coordinate system, *x*_1_ − *y*_1_ − *z*_1_ is a rectangular coordinate system, and the origin of coordinates of both coordinate systems is O point. In Fig 8, in order to calculate the distance $L_{J_{1}-J_{2}}$ between *J*_1_ and *J*_2_, it is necessary to transform the cylindrical coordinates where the yarn nodes are located during the braiding movement into rectangular coordinates.

According to the positional relationship shown as a in Fig 8:$\rho_{J_{1}}^{\rho -\theta -z}=R_{in}+\frac{3h}{4}$, $\rho_{J_{2}}^{\rho -\theta -z}=R_{in}+\frac{h}{2}$, $\theta_{J_{1}}^{\rho -\theta -z}=-\frac{180^{{}^{\circ}}}{M}$. Therefore, in the cylindrical coordinate system *ρ* − *θ* − *z*, the coordinates of *J*_1_ and *J*_2_ points are:

$$\left\{\begin{array}{c} J_{{}_{1}}^{R-\theta -z}=\left(R_{in}+\frac{3h}{4},-\frac{180^{{}^{\circ}}}{M},w_{h}\right)\\ J_{{}_{2}}^{R-\theta -z}=\left(R_{in}+\frac{h}{2},0,\frac{3w_{h}}{2}\right) \end{array}\right.$$ (1)

Since the cylindrical coordinate system *ρ* − *θ* − *z* and the rectangular coordinate system *x*_1_ − *y*_1_ − *z*_1_ have the same origin O, according to the positional relationship between the two coordinate systems, the coordinates of *J*_1_ and *J*_2_ points in the rectangular coordinate system *x*_1_ − *y*_1_ − *z*_1_ are:

$$\left\{\begin{array}{c} J_{{}_{1}}^{x_{1}-y_{1}-z_{1}}=\left(-(R_{in}+\frac{3h}{4})\text{sin}(\frac{180^{{}^{\circ}}}{M}),w_{h},(R_{in}+\frac{3h}{4})\text{cos}(\frac{180^{{}^{\circ}}}{M})\right)\\ J_{{}_{2}}^{x_{1}-y_{1}-z_{1}}=\left(0,\frac{3w_{h}}{2},R_{in}+\frac{h}{2}\right) \end{array}\right.$$ (2)

In Fig 8(a), in the rectangular coordinate system *x*_1_ − *y*_1_ − *z*_1_, the distance between *J*_1_ and *J*_2_ is:

$$L_{J_{1}-J_{2}}^{x_{1}-y_{1}-z_{1}}=\sqrt{{\left[(R_{in}+\frac{3h}{4})\text{sin}(\frac{180^{{}^{\circ}}}{M})\right]}^{2}+{\left[\frac{w_{h}}{2}\right]}^{2}+{\left[(R_{in}+\frac{3h}{4})\text{cos}(\frac{180^{{}^{\circ}}}{M})-R_{in}-\frac{h}{2}\right]}^{2}}$$ (3)

In Fig 8(b), in the cylindrical coordinate system *ρ* − *θ* − *z*, the coordinates of *A*_5_ and *A*_6_ points are:

$$\left\{\begin{array}{c} A_{{}_{5}}^{R-\theta -z}=\left(R_{in}+\frac{h}{4},-\frac{180^{{}^{\circ}}}{M},2w_{h}\right)\\ A_{{}_{6}}^{R-\theta -z}=\left(R_{in},0,\frac{3w_{h}}{2}\right) \end{array}\right.$$ (4)

Similarly, in the rectangular coordinate system *x*_1_ − *y*_1_ − *z*_1_, the coordinates of *A*_5_ and *A*_6_ points are:

$$\left\{\begin{array}{c} A_{{}_{5}}^{x_{1}-y_{1}-z_{1}}=\left(-(R_{in}+\frac{h}{4})\text{sin}(\frac{180^{{}^{\circ}}}{M}),2w_{h},(R_{in}+\frac{h}{4})\text{cos}(\frac{180^{{}^{\circ}}}{M})\right)\\ A_{{}_{6}}^{x_{1}-y_{1}-z_{1}}=\left(0,\frac{3w_{h}}{2},R_{in}\right) \end{array}\right.$$ (5)

In Fig 8(b), in the rectangular coordinate system *x*_1_ − *y*_1_ − *z*_1_, the distance between *A*_5_ and *A*_6_ is:

$$L_{A_{5}-A_{6}}^{x_{1}-y_{1}-z_{1}}=\sqrt{{\left[(R_{in}+\frac{h}{4})\text{sin}(\frac{180^{{}^{\circ}}}{M})\right]}^{2}+{\left[\frac{w_{h}}{2}\right]}^{2}+{\left[(R_{in}+\frac{h}{4})\text{cos}(\frac{180^{{}^{\circ}}}{M})-R_{in}\right]}^{2}}$$ (6)

Therefore, according to the positional relationship shown in Fig 8, the formula for calculating the braiding angle of the inner cell A can be obtained:

α1=arccos{h4[(Rin+3h4)sin(180°M)]2+[wh2]2+[(Rin+3h4)cos(180°M)−Rin−h2]2}α2=arccos{h4[(Rin+h4)sin180°M]2+[wh2]2+[(Rin+h4)cos180°M−Rin]2} (7)

**Fig 9:**
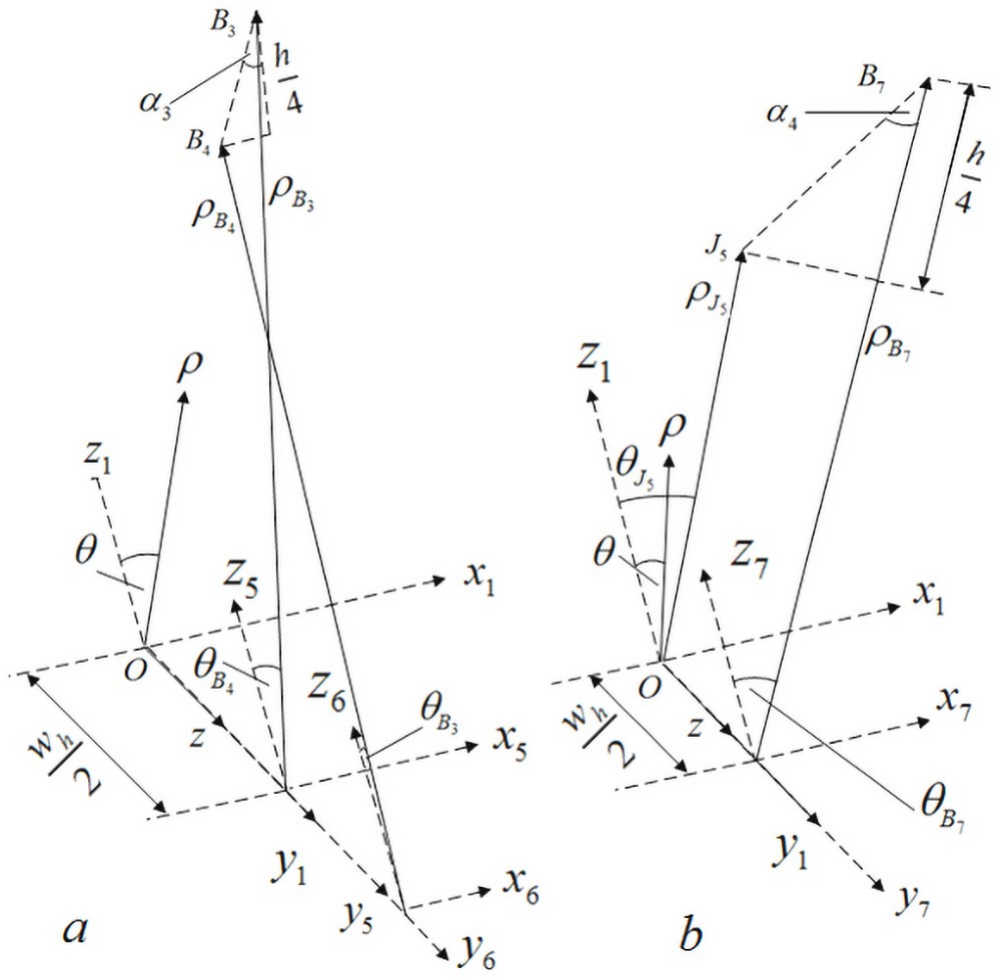
Calculation of braiding angle of internal cell B.

Similarly, according to the positional relationship shown in Fig 9, the formula for calculating the braiding angle of the inner cell B can be obtained:

α3=arccos{h4[Xα3]2+[wh2]2+[Routcos(360°M)−(Rin+3h4)cos(180°M)]2}α4=arccos{h4[Xα4]2+[wh2]2+[(Rin+h4)cos(180°M)−(Rin+h2)cos(360°M)]2} (8)

In formula (8),

$$\left\{\begin{array}{c} X_{\alpha_{3}}=R_{out}\text{cos}(\frac{360^{{}^{\circ}}}{M})-(R_{in}+\frac{3h}{4})\text{cos}(\frac{180^{{}^{\circ}}}{M})\\ X_{\alpha_{4}}=(R_{in}+\frac{h}{4})\text{cos}(\frac{180^{{}^{\circ}}}{M})-(R_{in}+\frac{h}{2})\text{cos}(\frac{360^{{}^{\circ}}}{M}) \end{array}\right..$$

**Fig 10:**
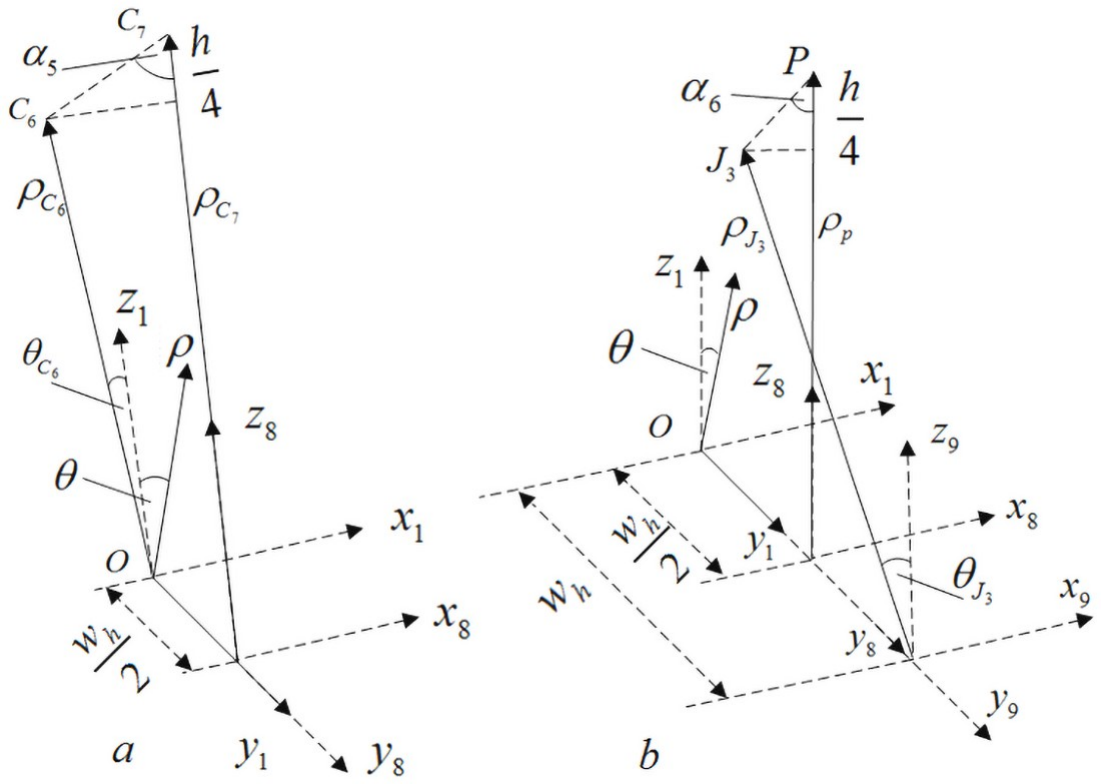
Calculation of braiding angle of internal cell C.

Similarly, according to the positional relationship shown in Fig 10, the formula for calculating the braiding angle of the inner cell C can be obtained:

α5=arccos{h4[(Rin+3h4)sin(180°M)−Rout]2+[wh2]2+[(Rin+3h4)cos(180°M)−Rout]2}α6=arccos{h4[(Rin+h4)sin(180°M)]2+[wh2]2+[(Rin+h2)−(Rin+h4)cos(180°M)]2} (9)

**Fig 11:**
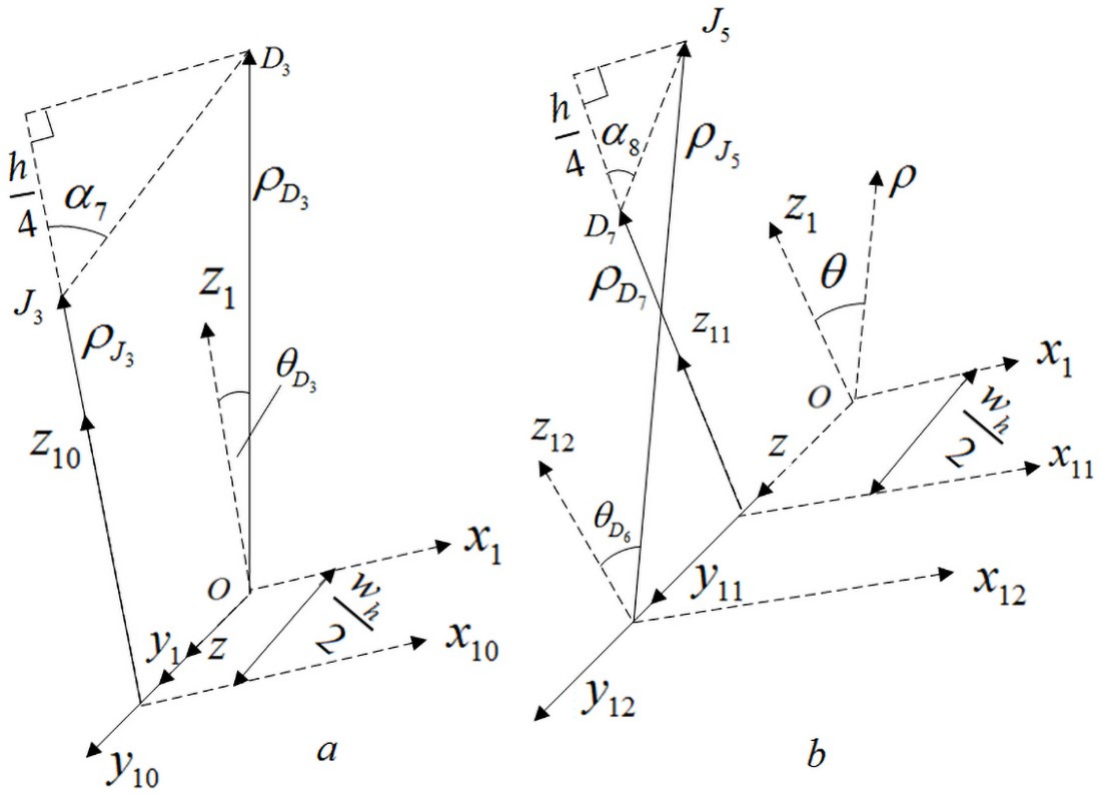
Calculation of braiding angle of internal cell D.

Similarly, according to the positional relationship shown in Fig 11, the formula for calculating the braiding angle of the inner cell D can be obtained:

α7=arccos{h4[(Rin+3h4)sin(180°M)]2+(wh2)2+[Rin+h2−(Rin+3h4)cos(180°M)]2}α8=arccos{h4[(Rin+h4)sin(180°M)]2+(wh2)2+[(Rin+h4)cos(180°M)−Rin]2} (10)

According to formulas (7)~(10), combined with Figs 8–11, it is found that there are relationships among the four internal cell braiding angles: *α*_1_ = *α*_7_ > *α*_2_ = *α*_8_, *α*_3_ = *α*_5_ > *α*_4_ = *α*_6_, that is, the braiding angles of the four inner cells gradually increase from inside to outside, that is, there are four different braiding angles in the four inner cells.

According to the braiding angle of inner cells, the formulas for calculating the total volume of inner cells $V_{in}^{1}$, the volume of fibers in inner cells *V*_1_ and the volume content of fibers *V*_*in*_ can be obtained:

$$\left\{\begin{array}{c} V_{in}^{1}=\frac{4\pi (R_{out}^{2}-R_{in}^{2})w_{h}}{M+N}\\ \begin{array}{c} V_{1}=\frac{S_{1}h}{\text{cos}\alpha_{1}}+\frac{S_{1}h}{\text{cos}\alpha_{2}}+\frac{S_{1}h}{\text{cos}\alpha_{3}}+\frac{S_{1}h}{\text{cos}\alpha_{4}}+4S_{2}h\\ V_{in}=\frac{V_{1}}{V_{in}^{1}} \end{array} \end{array}\right.$$ (11)

In formula (11), *S*_1_ is the cross-sectional area of braided yarn fiber bundle, and *S*_2_ is the cross-sectional area of axial yarn.

### (2) Parametric calculation of upper surface unit cell

**Fig 12:**
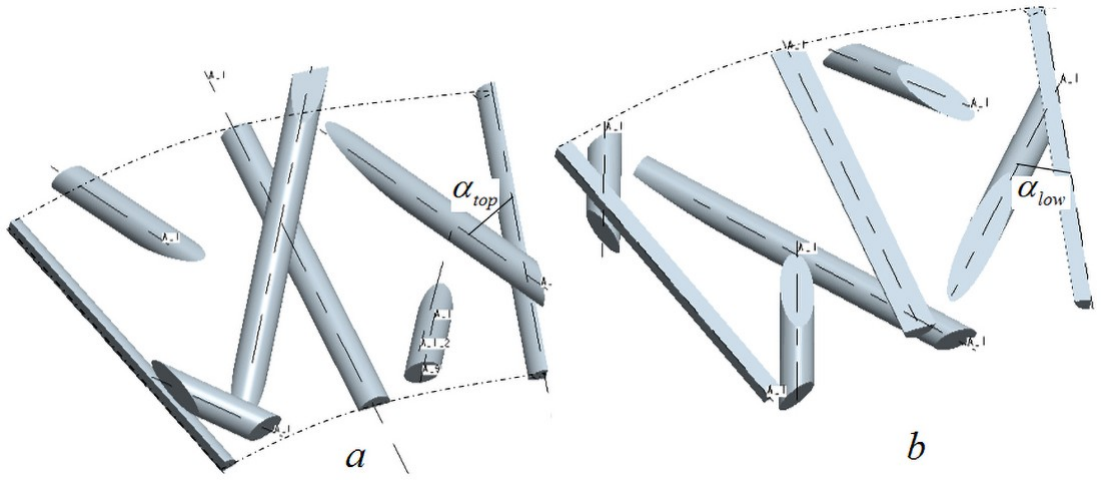
Positional relationship between upper and lower surface cells.

According to the positional relationship shown in Fig 12(a), the formula for calculating the braiding angle of the upper surface cell can be obtained:

αtop=arccos{h4ξ2+(wh2)2+[(Rin+h4)cos(360°M)−(Rin+3h4)cos(180°M)]2} (12)

In formula (12), $\xi_{top}=[(R_{in}+\frac{h}{4})\text{sin}(\frac{360^{{}^{\circ}}}{M})-(R_{in}+\frac{3h}{4})\text{sin}(\frac{180^{{}^{\circ}}}{M})]$.

According to the braiding angle of the upper surface cell, the formulas for calculating the total volume of upper surface cell $V_{top}^{2}$, the volume of fibers in upper surface cell *V*_2_ and the volume content of fibers *V*_*top*_ can be obtained:

$$\left\{\begin{array}{c} V_{top}^{2}=\frac{2\pi (R_{out}^{2}-R_{in}^{2})w_{h}}{M+N}\\ \begin{array}{c} V_{2}=\frac{S_{1}h}{\text{cos}\alpha_{top}}+\frac{S_{2}h}{2}\\ V_{top}=\frac{V_{2}}{V_{top}^{2}} \end{array} \end{array}\right.$$ (13)

In formula (13), *S*_1_ is the cross-sectional area of braided yarn fiber bundle and *S*_2_ is the cross-sectional area of axial yarn.

### (3) Parametric calculation of lower surface unit cell

According to the positional relationship shown in Fig 12(b), the formula for calculating the braiding angle of the lower surface cell can be obtained:

αlow=arccos{h4ξ2+(wh2)2+[(Rin+3h4)cos(360°M)−(Rin+h4)cos(180°M)]2} (14)

In formula (14), $\xi =[(R_{in}+\frac{3h}{4})\text{sin}(\frac{360^{{}^{\circ}}}{M})-(R_{in}+\frac{h}{4})\text{sin}(\frac{180^{{}^{\circ}}}{M})]$.

According to the braiding angle of the lower surface cell, the formulas for calculating the total volume of lower surface cell $V_{low}^{3}$, the volume of fibers in lower surface cell *V*_3_ and the volume content of fibers *V*_*low*_ can be obtained [5–7]:

$$\left\{\begin{array}{c} V_{low}^{3}=\frac{2\pi (R_{out}^{2}-R_{in}^{2})w_{h}}{M}\\ \begin{array}{c} V_{3}=\frac{S_{1}h}{\text{cos}\alpha_{low}}+\frac{S_{2}h}{2}\\ V_{low}=\frac{V_{3}}{V_{low}^{3}} \end{array} \end{array}\right.$$ (15)

In formula (15), *S*_1_ is the cross-sectional area of braided yarn fiber bundle and *S*_2_ is the cross-sectional area of axial yarn.

The total fiber volume content of 3D annular braided material is the sum of the product of the fiber volume content of each cell and the percentage of the whole cell volume occupied by each cell [5–7]:

$$V_{f}=\kappa_{in}V_{in}+\kappa_{top}V_{top}+\kappa_{low}V_{low}$$ (16)

In formula (16), *κ*_*in*_, *κ*_*top*_ and *κ*_*low*_ are the percentage of the inner, upper and lower surface cells in the whole cell volume. *V*_*in*_, *V*_*top*_ and *V*_*low*_ are the volume contents of fibers on the inner, upper and lower surfaces.

T300 carbon fiber is used for braiding yarn in this paper. The matrix is TDE-86 epoxy resin. The mass calculation formula of the 3D annular braided material cell is as follows:

$$m=\left(V_{1}\text{+}V_{2}\text{+}V_{3}\right)\rho_{1}+\left(V_{in}^{1}+V_{out}^{2}+V_{low}^{3}-V_{1}-V_{2}-V_{3}\right)\rho_{2}$$ (17)

In formula (17), *m* is the mass of the 3D annular braided material cells, *ρ*_1_ is the carbon fiber density, and *ρ*_2_ is the matrix density.

## 4. Discussion on cell parameters of 3D circular braided materials

**Fig 13:**
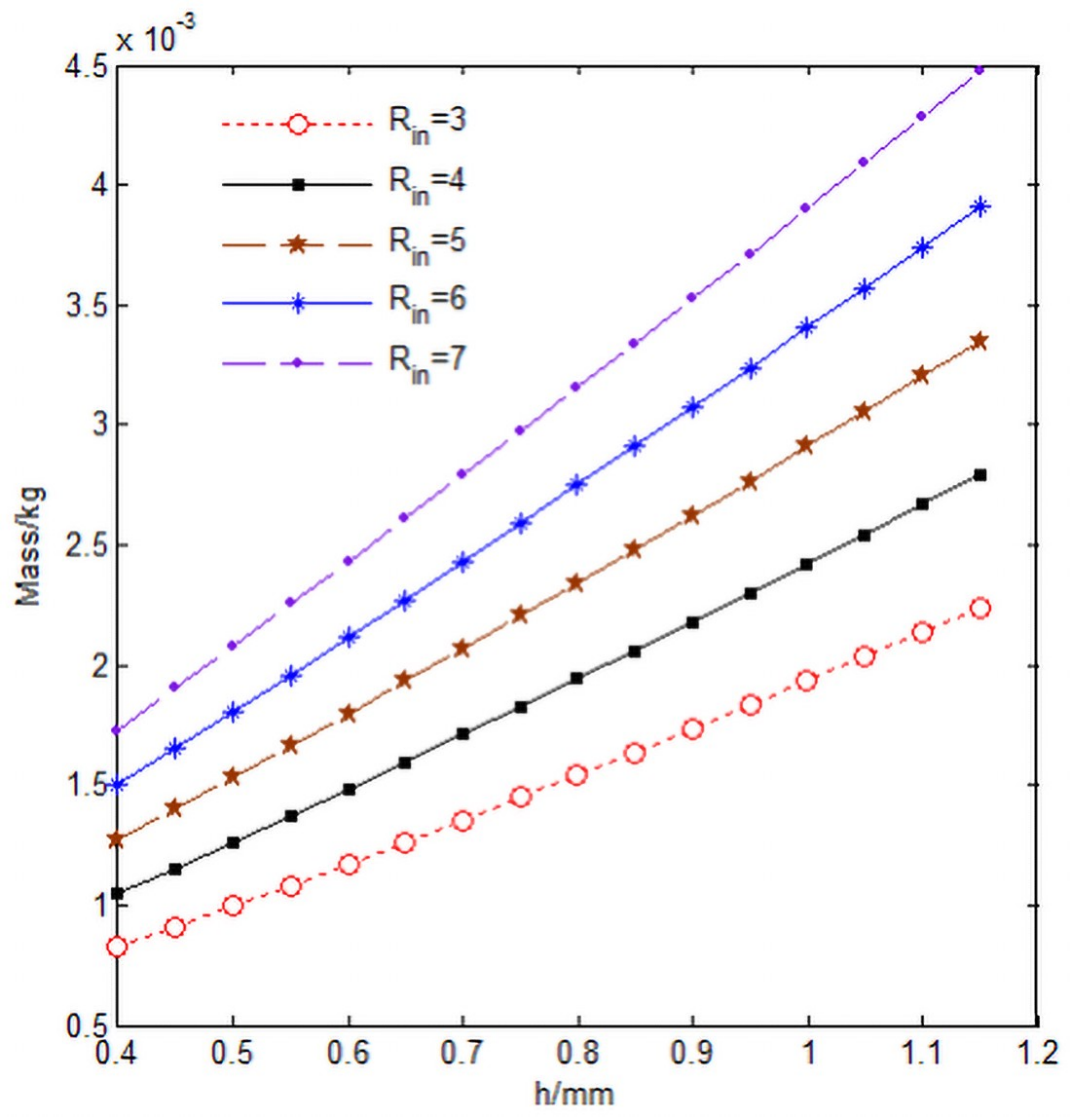
Relationship curve of cell mass *m*, knuckle length h and cell inner diameter *R*_*in*_.

Fig 13 shows the relationship between the mass m of the 3D annular braided material cells and the knuckle length h, and the values of the parameters in the Fig 13 are shown in Table 4.

**Table 4:** Braiding parameters.

Parameter	Size
number of braiding yarns	*M* = 40
number of axial yarns	*N* = 40
the height of inner daughter cell	*w*_*h*_ = 0.4*mm*
the carbon fiber density	*ρ*_1_ = 1.8*kg*/*mm*^3^
the matrix density	*ρ*_2_ = 0.9*kg*/*mm*^3^

It can be seen from Fig 13 that with the increase of the knuckle length h and the inner diameter *R*_*in*_ of the cell, the mass m of the cell increases proportionally, which is mainly due to the increase of the knuckle length h and the inner diameter *R*_*in*_ of the cell, which increases the size of the cell in the diameter direction and the circumferential direction respectively, resulting in the increase of the volume of the cell and the mass of the cell (Fig 13 program source code in S1 File).

**Fig 14:**
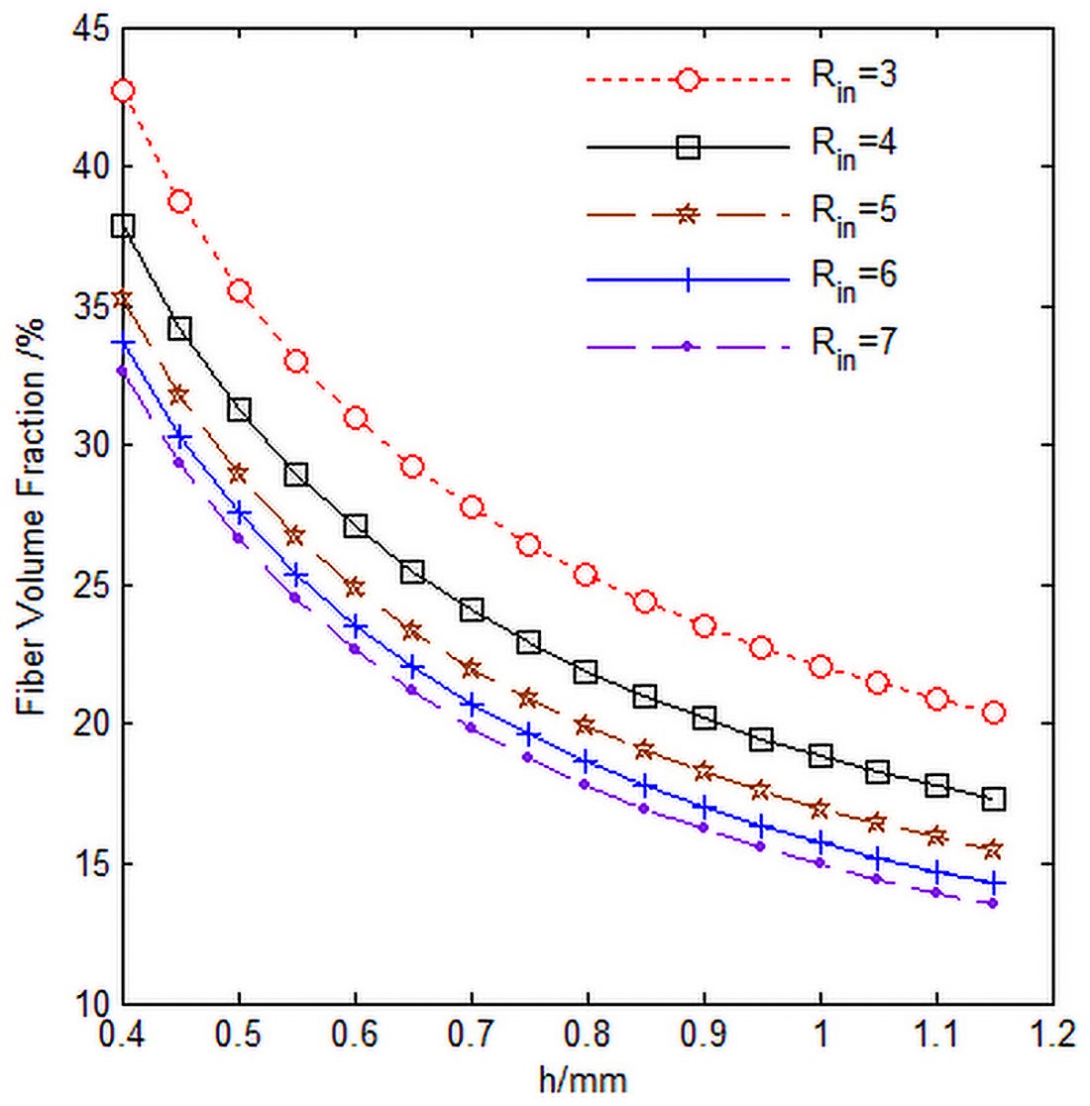
Relationship curve of fiber volume content *V*_*f*_, knuckle length h and cell inner diameter *R*_*in*_.

The relationship between the total fiber volume content *V*_*f*_ of the 3D annular braided material and the knuckle length h is shown in Fig 14, and the values of the parameters in the Fig 14 are shown in Table 4.

It can be seen from Fig 14 that with the increase of the knuckle length h and the cell inner diameter *R*_*in*_, the total cell fiber volume content *V*_*f*_ decreases, which is mainly due to the increase of the knuckle length and the cell inner diameter, which increases the size of the cell in the diameter direction and the circumferential direction, increases the gap between the braided fibers and fills the gap between the braided fibers with the matrix, resulting in the decrease of the total cell fiber volume content (Fig 14 program source code in S1 File).

**Fig 15:**
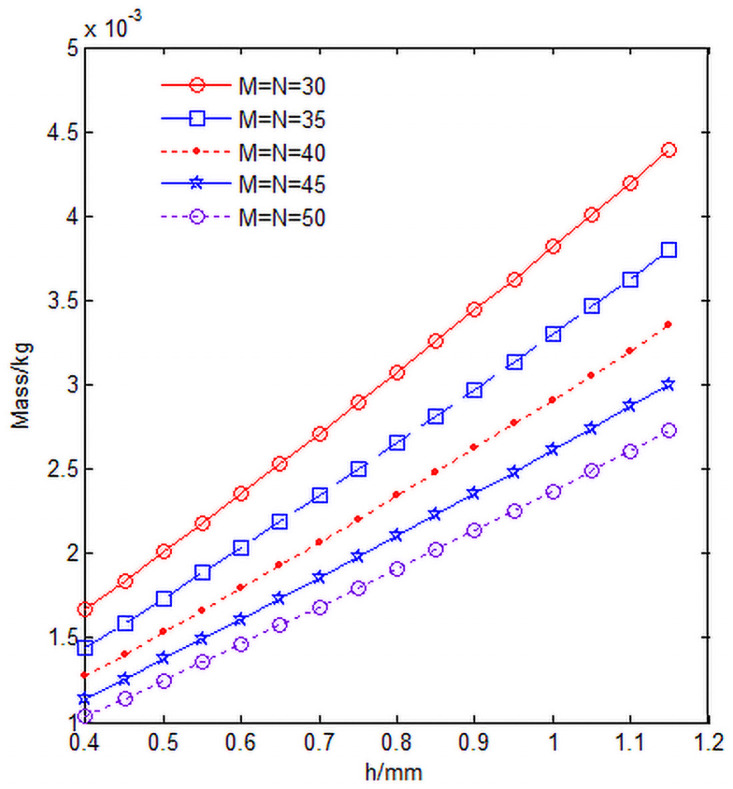
Relationship curve of unit cell mass m, knuckle length h and yarn number.

The relationship between the mass m, knuckle length h, radial yarn number M and axial yarn number N of the 3D annular braiding material unit cell is shown in Fig 15, in which the unit cell inner diameter *R*_*in*_ = 5*mm*.

It can be seen from Fig 15 that with the increase of the knuckle length, the mass of the single cell increases proportionally. With the increase of radial yarn number M and axial yarn number N, the mass of cells decreases proportionally, which is mainly due to the increase of radial yarn number M and axial yarn number N, and the decrease of the angle of fan-shaped area where cells are located, which leads to the smaller size and volume of cells in the circumferential direction, resulting in the smaller mass of cells (Fig 15 program source code in S2 File).

**Fig 16:**
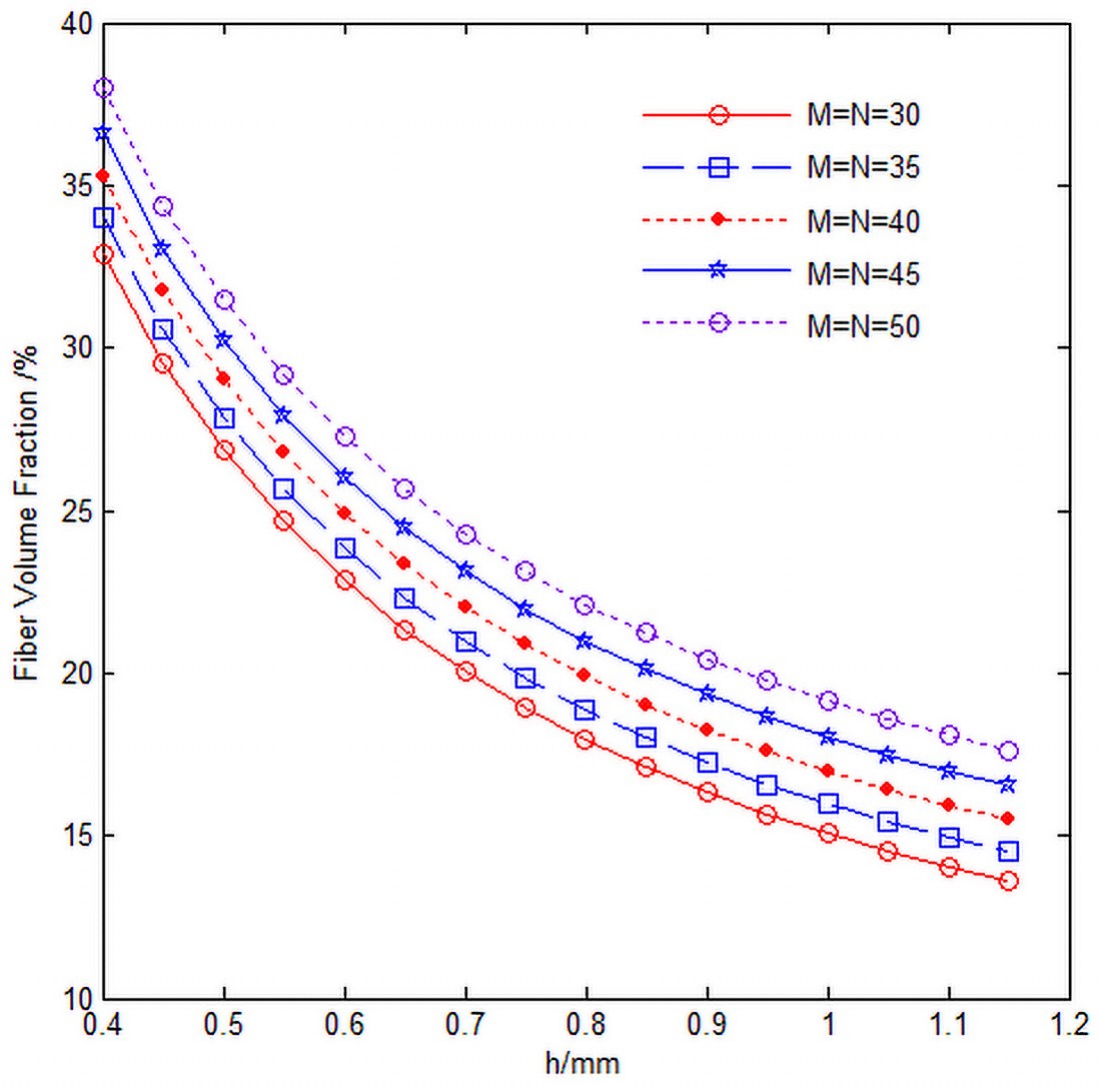
Relationship curve of fiber volume content *V*_*f*_, knuckle length h and yarn number.

The relationship among fiber volume content *V*_*f*_, knuckle length h, radial yarn number M and axial yarn number N of 3D circular braiding material is shown in Fig 16, in which the inner diameter of single cell is *R*_*in*_ = 5*mm*.

It can be seen from Fig 16 that with the increase of knuckle length, the total fiber volume content of single cell decreases, and with the increase of radial yarn number M and axial yarn number N, it is mainly due to the increase of radial yarn number M and axial yarn number N, the angle of fan-shaped area where single cell is located decreases, and the gap between braided fibers decreases, so the total fiber volume content of single cell increases (Fig 16 program source code in S2 File).

**Fig 17:**
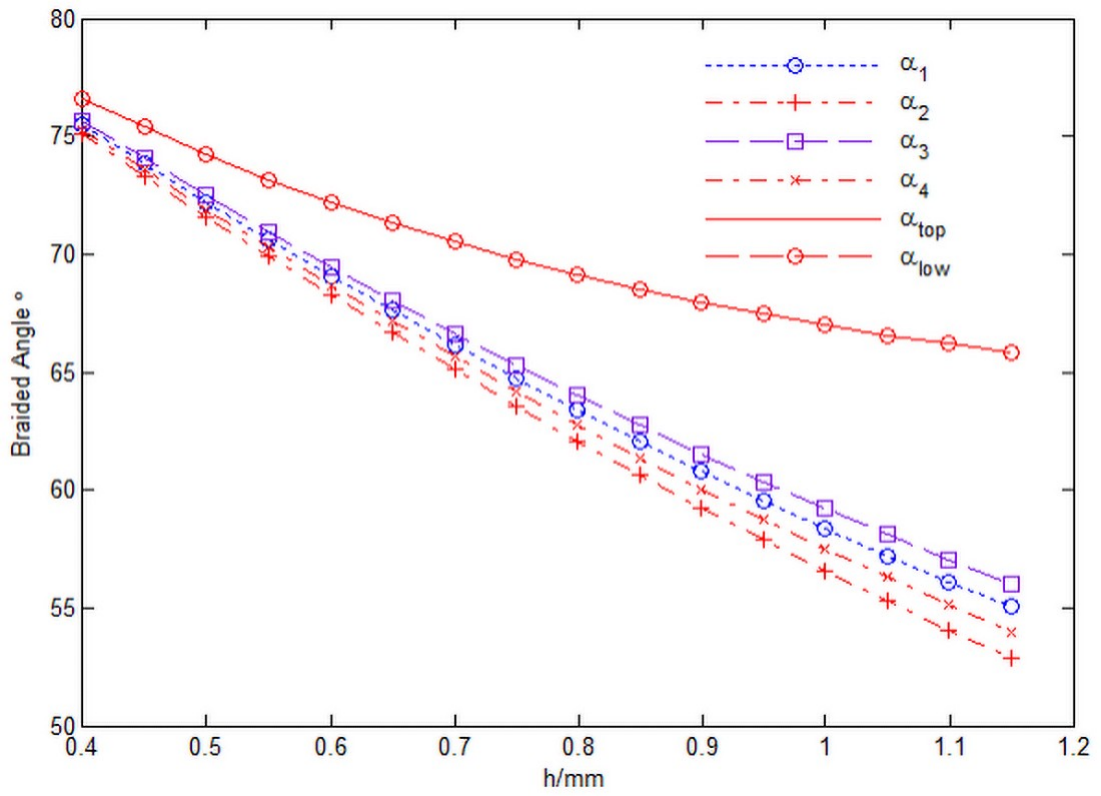
Relationship curve between braiding angle *α* and knuckle length h.

The relationship between braiding angle *α* and knuckle length h of 3D circular braiding material is shown in Fig 17. In that Fig 17, the cell inner diameter *R*_*in*_ = 5*mm*, the height of inner daughter cell *w*_*h*_ = 0.4*mm*, the radial yarn number M = 50 and the axial yarn number N = 50.

It can be seen from Fig 17 that the braiding angles of four internal cells are close to each other, which is satisfied *α*_3_ > *α*_1_ > *α*_4_ > *α*_2_. With the increase of the knuckle length, the braiding angles of each cell decrease, and the braiding angles of the four internal cells decrease the most. With the increase of the knuckle length, the braiding angle of the upper and lower surface cells decreases slightly, that is, *α*_*top*_ ≈ *α*_*low*_ (Fig 17 program source code in S3 File).

## 5. Conclusion

1. On the basis of analyzing the movement law of 3D five-direction circular braiding yarn, the minimum research unit of 3D five-direction circular braiding composite material is divided into three forms: upper surface unit cell, lower surface unit cell, internal unit cell. Through the coordinate transformation of yarn node position during braiding movement, the parametric calculation formulas of braiding angle in various cell models are obtained. According to the positional relationship of radial yarn, axial yarn and matrix in the three cell models, the parametric calculation formulas of geometric characteristics such as matrix volume, fiber volume, fiber volume content and mass of the three cell models are derived by using the braiding angle. Parametric formula can be used to analyze the influence of yarn position change on the physical properties of single cell under different braiding parameters.
2. Through the analysis of different cell braiding angle formulas, it is found that there are four different sizes of braiding angles in the inner cell, which is satisfied *α*_1_ = *α*_7_ > *α*_2_ = *α*_8_, *α*_3_ = *α*_5_ > *α*_4_ = *α*_6_. The braiding angle in the inner cell gradually increases from inside to outside along the braiding direction. The braiding angles of upper and lower surface cells are approximately equal, that is, *α*_*top*_ ≈ *α*_*low*_.
3. The results of parametric analysis show that with the increase of the knuckle length, the braiding angles of each cell decrease, and the braiding angles of the four inner cells decrease greatly, while the braiding angles of the upper and lower surfaces decrease slightly. With the increase of the knuckle length and the inner diameter of the cell, the mass of the cell increases proportionally, while the total fiber volume content of the cell decreases. With the increase of radial yarn number and axial yarn number, the unit cell mass decreases in direct proportion, and the unit cell total fiber volume content increases. The results of this paper directly reflect the influence of the knuckle length on the physical properties of the cell.
4. Using design variables such as cell inner diameter *R*_*in*_, cell outer diameter *R*_*out*_, radial yarn number M, axial yarn number N, height of inner daughter cell *w*_*h*_, knuckle length h, cross-sectional area of braiding yarn *S*_1_ and cross-sectional area of axial yarn *S*_2_, the position coordinates of yarn nodes in a cell can be quickly calculated, and the yarn space motion trajectory can be obtained by connecting the beginning and the end of the nodes, so the modeling efficiency of parametric model can be greatly improved by using the research results of this paper.

## Supporting information

S1 File Figs 13 and 14 program source code. (DOCX)

S2 File Figs 15 and 16 program source code. (DOCX)

S3 File Fig 17 program source code. (DOCX)

## References

[1] Ma WS, Feng W. variable microstructural unit-cell geometrical analysis model of 3D braided tubular composites and components. Acta Materiae Compositae Sinica. 2005;22(5):162–170.

[2] Chen L, Li JL, Li XM. analysis of 4-step 3-D tubular braiding structures. Acta Materiae Compositae Sinica. 2003;20(2):76–80.

[3] Kang HR, Shan ZD, Zang Y, Liu F. progressive damage analysis and strength properties of fiber-bar composites reinforced by three-dimensional weaving under uniaxial tension. Composite Structures. 2016;141:264–28. 10.1016/j.compstruct.2016.01.050

[4] Sun XK. micro-geometry of 3-D braided tubular preform. Journal of composite materials.2004;38(9):791–798. 10.1177/0021998304042147.

[5] Lu ZX, Liu ZG, Mai HC, Chen ZR. numerical prediction of strength for 3D braided composites. Journal of Beijing University of Aeronautics and Astronautics. 2002;28(5):563–565. 10.1007/s11766-996-0015-2.

[6] Lu ZX, Yang ZY, Liu ZG. development of investigation into mechanical behaviour of three dimensional braided composites. Acta Materiae Compositae Sinica. 2004;21(2):1–6.

[7] Lu ZX, Yang ZY, Liu ZG. geometrical characteristics of structural model for 3-D braided composites. Journal of Beijing University of Aeronautics and Astronautics. 2006; 32(1):92–95. 10.1677/jme.1.02008.

[8] Zhang C, Xu XW. application of three unit-cells models on mechanical analysis of 3D five-directional and full five-directional braided composites. Appl Compos Mater. 2013;20:803–825. 10.1007/s10443-012-93090.

[9] Zhang C. finite element analysis of the damage mechanism of 3D braided composites under high-velocity impact. J Mater Sci. 2017;52:4658–4674. 10.1007/s10853-016-0709-7.

[10] Zhang F, Liu ZG, Wu Z, Tao GQ. a new scheme and micro structural model for 3D full 5-directional braided composites. Chinese Journal of Aeronautics. 2010;23:61–67. 10.1016/S1000-9361(09)60188-6.

[11] Li DS, Lu ZX, Chen L, Li JL. microstructure analysis and prediction of the elastic properties of 3D and 5-D tubular braided composites. Acta Aeronautica Et Astronautica Sinica. 2007;28(1):1223–129. 10.1016/S1001-6058(07)-60030-4.

[12] Li L, Aliabadi MH. elastic property prediction and damage mechanics analysis of 3D braided composite. Theoretical and Applied Fracture Mechanics.2019;104:1–16. 10.1016/j.tafmec.2019.102338.

[13] Xu K, Xu XW. finite element analysis of mechanical properties of 3D five-directional braided composites. Materials Science and Engineering A.2008;487:499–509. 10.1016/j.msea.2007.10.030.

[14] Alpyildiz T. 3D geometrical modelling of tubular braids. Textile Research Journal. 2012; 82(5):443–453. 10.1177/0040517511427969.

[15] Chen L, Tao XM, Choy CL. on the microstructure of three-dimensional braided preforms. Composites Science and Technology.1999;59:391–404. 10.1016/S0266-3538(98)00079-7.

[16] Verpoest I, Stepan V. virtual textile composites software wisetex: integration with micro-mechanical, permeability and structural analysis. Composites Science and Technology.2005;65:2563–2574. 10.1016/j.compscitech.2005.05.031.

[17] Fang GD, Liang J, Wang BL. progressive damage and nonlinear analysis of 3D four-directional braided composites under unidirectional tension. Composite Structures.2009; 89:126–133. 10.1016/j.compstruct.2008.07.016.

[18] Fang GD, Liang J, Wang BL, Y Wang. investigation on the compressive properties of the three dimensional four-directional braided composites. Composite Structures. 2011; 93:392–405. 10.1016/j.compstruct.2010.09.002.

[19] Fang GD, Liang J. a review of numerical modeling of three dimensional braided textile composites. Journal of Composite Materials. 2015;45(23):2415–2436. 10.1177/0021998311401093

[20] Wang YB, Liu ZG, Liu N, Hu L, Wei YC, Ou JJ. a new geometric modelling approach for 3D braided tubular composites base on free form deformation. Composite Structures. 2016;136:75–85.

[21] Wang YB, Liu ZG, Hu L, Wu Z. predicting the elastic modulus of 3D braided composite tubes using geometrical mapping approach.21st International Conference on Composite Materials, Xi’an. 20-25th August (2017).

