# Supplementary material for: Parametric characteristics analysis of three cells in 3D and five-directional annular braided composites
Source: PLoS One. 2021 Aug 4;16(8):e0254691. doi: 10.1371/journal.pone.0254691 (PMC8336883; doi:10.1371/journal.pone.0254691)

**%%%Fig15-16 program source code**

clc;clear all;

S_1=0.031;S_2=0.0081;w_h=0.4;

h=0.4:0.05:1.2;R_in=5;R_out=R_in+h;M=50;N=0;

rou_1=1.8e-3;%%% 碳纤维密度，单位：g/mm3

rou_2=0.9e-3;%%% 基体密度，单位：g/mm3

for i=1:(length(h)-1)

A_1(i)=sqrt(((R_in+0.75.*h(i))*sin(pi/(M+N))).^2+(w_h/2).^2+(((R_in+0.75*h(i))*cos(pi/(M+N)))-R_in-0.5.*h(i)).^2);

aerfa_1(i)=acos(0.25*h(i)/A_1(i));

aerfa_11(i)=360/(2*pi)*acos(0.25*h(i)/A_1(i));

A_2(i)=sqrt(((R_in+0.25*h(i))*sin(pi/(M+N))).^2+(w_h/2).^2+(((R_in+0.25*h(i))*cos(pi/(M+N)))-R_in).^2);

aerfa_2(i)=acos(0.25*h(i)/A_2(i));%%%弧度

aerfa_22(i)=360/(2*pi)*acos(0.25*h(i)/A_2(i));%%角度

A_3(i)=sqrt(((R_in+1.*h(i))*sin(2*pi/(M+N))-(R_in+0.75.*h(i))*sin(pi/(M+N))).^2+(w_h/2).^2+((R_in+1.*h(i))*cos(2*pi/(M+N))-(R_in+0.75.*h(i))*cos(pi/(M+N))).^2);

aerfa_3(i)=acos(0.25*h(i)/A_3(i));%%%弧度

aerfa_33(i)=360/(2*pi)*acos(0.25*h(i)/A_3(i));%%角度

A_4(i)=sqrt((((R_in+0.25*h(i))*sin(pi/(M+N)))-(R_in+0.25*h(i))*sin(2*pi/(M+N)))^2+(w_h/2)^2+(((R_in+0.25*h(i))*cos(pi/(M+N)))-(R_in+0.25*h(i))*cos(2*pi/(M+N)))^2);

aerfa_4(i)=acos(0.25*h(i)/A_4(i));%%%弧度

aerfa_44(i)=360/(2*pi)*acos(0.25*h(i)/A_4(i));%%角度

A_top(i)=sqrt((((R_in+0.25*h(i))*sin(2*pi/(M+N)))-(R_in+0.75*h(i))*sin(pi/(M+N)))^2+(w_h/2)^2+(((R_in+0.25*h(i))*cos(2*pi/(M+N)))-(R_in+0.75*h(i))*cos(pi/(M+N)))^2);

aerfa_top(i)=acos(0.25*h(i)/A_top(i));%%%弧度

aerfa_topp(i)=360/(2*pi)*acos(0.25*h(i)/A_top(i));%%角度

A_low(i)=sqrt((((R_in+0.75*h(i))*sin(2*pi/(M+N)))-(R_in+0.25*h(i))*sin(pi/(M+N)))^2+(w_h/2)^2+(((R_in+0.75*h(i))*cos(2*pi/(M+N)))-(R_in+0.25*h(i))*cos(pi/(M+N)))^2);

aerfa_low(i)=acos(0.25*h(i)/A_low(i));%%%弧度

aerfa_loww(i)=360/(2*pi)*acos(0.25*h(i)/A_low(i));%%角度

V_in1(i)=4*pi*((R_in+h(i)).^2-(R_in).^2)*w_h/(M+N);

V_1(i)=S_1*h(i)/cos(aerfa_1(i))+S_1*h(i)/cos(aerfa_2(i))+S_1*h(i)/cos(aerfa_3(i))+S_1*h(i)/cos(aerfa_4(i))+4*S_2*h(i);

kuta_in(i)=V_1(i)/V_in1(i);

V_top2(i)=2*pi*((R_in+h(i)).^2-(R_in).^2)*w_h/(M+N);

V_2(i)=S_1*h(i)/cos(aerfa_top(i))+S_2*h(i)/2;

kuta_top(i)=V_2(i)/V_top2(i);

V_low3(i)=2*pi*((R_in+h(i)).^2-(R_in).^2)*w_h/(M+N);

V_3(i)=S_1*h(i)/cos(aerfa_low(i))+S_2*h(i)/2;

kuta_low(i)=V_3(i)/V_low3(i);

V_f(i)=(0.5*kuta_in(i)+0.25*kuta_top(i)+0.25*kuta_low(i))*100;

VV(i)=V_1(i)+V_2(i)+V_3(i);

WW(i)=V_in1(i)+V_top2(i)+V_low3(i)-VV(i);

mass(i)=rou_1.*VV(i)+rou_2.*WW(i);

end

hold on

subplot(1,2,1)

hold on

plot(0.4:0.05:1.15,mass,'*')

xlabel('h/mm');ylabel('Mass/kg');

hold on

subplot(1,2,2)

hold on

plot(0.4:0.05:1.15,V_f,'+')

xlabel('h/mm');ylabel('Fiber Volume Fraction /%');

hold on

clc;clear all;

S_1=0.031;S_2=0.0081;w_h=0.4;

h=0.4:0.05:1.2;R_in=5;R_out=R_in+h;M=45;N=0;

rou_1=1.8e-3;%%% 碳纤维密度，单位：g/mm3

rou_2=0.9e-3;%%% 基体密度，单位：g/mm3

for i=1:(length(h)-1)

A_1(i)=sqrt(((R_in+0.75.*h(i))*sin(pi/(M+N))).^2+(w_h/2).^2+(((R_in+0.75*h(i))*cos(pi/(M+N)))-R_in-0.5.*h(i)).^2);

aerfa_1(i)=acos(0.25*h(i)/A_1(i));

aerfa_11(i)=360/(2*pi)*acos(0.25*h(i)/A_1(i));

A_2(i)=sqrt(((R_in+0.25*h(i))*sin(pi/(M+N))).^2+(w_h/2).^2+(((R_in+0.25*h(i))*cos(pi/(M+N)))-R_in).^2);

aerfa_2(i)=acos(0.25*h(i)/A_2(i));%%%弧度

aerfa_22(i)=360/(2*pi)*acos(0.25*h(i)/A_2(i));%%角度

A_3(i)=sqrt(((R_in+1.*h(i))*sin(2*pi/(M+N))-(R_in+0.75.*h(i))*sin(pi/(M+N))).^2+(w_h/2).^2+((R_in+1.*h(i))*cos(2*pi/(M+N))-(R_in+0.75.*h(i))*cos(pi/(M+N))).^2);

aerfa_3(i)=acos(0.25*h(i)/A_3(i));%%%弧度

aerfa_33(i)=360/(2*pi)*acos(0.25*h(i)/A_3(i));%%角度

A_4(i)=sqrt((((R_in+0.25*h(i))*sin(pi/(M+N)))-(R_in+0.25*h(i))*sin(2*pi/(M+N)))^2+(w_h/2)^2+(((R_in+0.25*h(i))*cos(pi/(M+N)))-(R_in+0.25*h(i))*cos(2*pi/(M+N)))^2);

aerfa_4(i)=acos(0.25*h(i)/A_4(i));%%%弧度

aerfa_44(i)=360/(2*pi)*acos(0.25*h(i)/A_4(i));%%角度

A_top(i)=sqrt((((R_in+0.25*h(i))*sin(2*pi/(M+N)))-(R_in+0.75*h(i))*sin(pi/(M+N)))^2+(w_h/2)^2+(((R_in+0.25*h(i))*cos(2*pi/(M+N)))-(R_in+0.75*h(i))*cos(pi/(M+N)))^2);

aerfa_top(i)=acos(0.25*h(i)/A_top(i));%%%弧度

aerfa_topp(i)=360/(2*pi)*acos(0.25*h(i)/A_top(i));%%角度

A_low(i)=sqrt((((R_in+0.75*h(i))*sin(2*pi/(M+N)))-(R_in+0.25*h(i))*sin(pi/(M+N)))^2+(w_h/2)^2+(((R_in+0.75*h(i))*cos(2*pi/(M+N)))-(R_in+0.25*h(i))*cos(pi/(M+N)))^2);

aerfa_low(i)=acos(0.25*h(i)/A_low(i));%%%弧度

aerfa_loww(i)=360/(2*pi)*acos(0.25*h(i)/A_low(i));%%角度

V_in1(i)=4*pi*((R_in+h(i)).^2-(R_in).^2)*w_h/(M+N);

V_1(i)=S_1*h(i)/cos(aerfa_1(i))+S_1*h(i)/cos(aerfa_2(i))+S_1*h(i)/cos(aerfa_3(i))+S_1*h(i)/cos(aerfa_4(i))+4*S_2*h(i);

kuta_in(i)=V_1(i)/V_in1(i);

V_top2(i)=2*pi*((R_in+h(i)).^2-(R_in).^2)*w_h/(M+N);

V_2(i)=S_1*h(i)/cos(aerfa_top(i))+S_2*h(i)/2;

kuta_top(i)=V_2(i)/V_top2(i);

V_low3(i)=2*pi*((R_in+h(i)).^2-(R_in).^2)*w_h/(M+N);

V_3(i)=S_1*h(i)/cos(aerfa_low(i))+S_2*h(i)/2;

kuta_low(i)=V_3(i)/V_low3(i);

V_f(i)=(0.5*kuta_in(i)+0.25*kuta_top(i)+0.25*kuta_low(i))*100;

VV(i)=V_1(i)+V_2(i)+V_3(i);

WW(i)=V_in1(i)+V_top2(i)+V_low3(i)-VV(i);

mass(i)=rou_1.*VV(i)+rou_2.*WW(i);

end

hold on

subplot(1,2,1)

hold on

plot(0.4:0.05:1.15,mass,'*')

xlabel('h/mm');ylabel('Mass/kg');

hold on

subplot(1,2,2)

hold on

plot(0.4:0.05:1.15,V_f,'+')

xlabel('h/mm');ylabel('Fiber Volume Fraction /%');

hold on

clc;clear all;

S_1=0.031;S_2=0.0081;w_h=0.4;

h=0.4:0.05:1.2;R_in=5;R_out=R_in+h;M=40;N=0;

rou_1=1.8e-3;%%% 碳纤维密度，单位：g/mm3

rou_2=0.9e-3;%%% 基体密度，单位：g/mm3

for i=1:(length(h)-1)

A_1(i)=sqrt(((R_in+0.75.*h(i))*sin(pi/(M+N))).^2+(w_h/2).^2+(((R_in+0.75*h(i))*cos(pi/(M+N)))-R_in-0.5.*h(i)).^2);

aerfa_1(i)=acos(0.25*h(i)/A_1(i));

aerfa_11(i)=360/(2*pi)*acos(0.25*h(i)/A_1(i));

A_2(i)=sqrt(((R_in+0.25*h(i))*sin(pi/(M+N))).^2+(w_h/2).^2+(((R_in+0.25*h(i))*cos(pi/(M+N)))-R_in).^2);

aerfa_2(i)=acos(0.25*h(i)/A_2(i));%%%弧度

aerfa_22(i)=360/(2*pi)*acos(0.25*h(i)/A_2(i));%%角度

A_3(i)=sqrt(((R_in+1.*h(i))*sin(2*pi/(M+N))-(R_in+0.75.*h(i))*sin(pi/(M+N))).^2+(w_h/2).^2+((R_in+1.*h(i))*cos(2*pi/(M+N))-(R_in+0.75.*h(i))*cos(pi/(M+N))).^2);

aerfa_3(i)=acos(0.25*h(i)/A_3(i));%%%弧度

aerfa_33(i)=360/(2*pi)*acos(0.25*h(i)/A_3(i));%%角度

A_4(i)=sqrt((((R_in+0.25*h(i))*sin(pi/(M+N)))-(R_in+0.25*h(i))*sin(2*pi/(M+N)))^2+(w_h/2)^2+(((R_in+0.25*h(i))*cos(pi/(M+N)))-(R_in+0.25*h(i))*cos(2*pi/(M+N)))^2);

aerfa_4(i)=acos(0.25*h(i)/A_4(i));%%%弧度

aerfa_44(i)=360/(2*pi)*acos(0.25*h(i)/A_4(i));%%角度

A_top(i)=sqrt((((R_in+0.25*h(i))*sin(2*pi/(M+N)))-(R_in+0.75*h(i))*sin(pi/(M+N)))^2+(w_h/2)^2+(((R_in+0.25*h(i))*cos(2*pi/(M+N)))-(R_in+0.75*h(i))*cos(pi/(M+N)))^2);

aerfa_top(i)=acos(0.25*h(i)/A_top(i));%%%弧度

aerfa_topp(i)=360/(2*pi)*acos(0.25*h(i)/A_top(i));%%角度

A_low(i)=sqrt((((R_in+0.75*h(i))*sin(2*pi/(M+N)))-(R_in+0.25*h(i))*sin(pi/(M+N)))^2+(w_h/2)^2+(((R_in+0.75*h(i))*cos(2*pi/(M+N)))-(R_in+0.25*h(i))*cos(pi/(M+N)))^2);

aerfa_low(i)=acos(0.25*h(i)/A_low(i));%%%弧度

aerfa_loww(i)=360/(2*pi)*acos(0.25*h(i)/A_low(i));%%角度

V_in1(i)=4*pi*((R_in+h(i)).^2-(R_in).^2)*w_h/(M+N);

V_1(i)=S_1*h(i)/cos(aerfa_1(i))+S_1*h(i)/cos(aerfa_2(i))+S_1*h(i)/cos(aerfa_3(i))+S_1*h(i)/cos(aerfa_4(i))+4*S_2*h(i);

kuta_in(i)=V_1(i)/V_in1(i);

V_top2(i)=2*pi*((R_in+h(i)).^2-(R_in).^2)*w_h/(M+N);

V_2(i)=S_1*h(i)/cos(aerfa_top(i))+S_2*h(i)/2;

kuta_top(i)=V_2(i)/V_top2(i);

V_low3(i)=2*pi*((R_in+h(i)).^2-(R_in).^2)*w_h/(M+N);

V_3(i)=S_1*h(i)/cos(aerfa_low(i))+S_2*h(i)/2;

kuta_low(i)=V_3(i)/V_low3(i);

V_f(i)=(0.5*kuta_in(i)+0.25*kuta_top(i)+0.25*kuta_low(i))*100;

VV(i)=V_1(i)+V_2(i)+V_3(i);

WW(i)=V_in1(i)+V_top2(i)+V_low3(i)-VV(i);

mass(i)=rou_1.*VV(i)+rou_2.*WW(i);

end

hold on

subplot(1,2,1)

hold on

plot(0.4:0.05:1.15,mass,'*')

xlabel('h/mm');ylabel('Mass/kg');

hold on

subplot(1,2,2)

hold on

plot(0.4:0.05:1.15,V_f,'+')

xlabel('h/mm');ylabel('Fiber Volume Fraction /%');


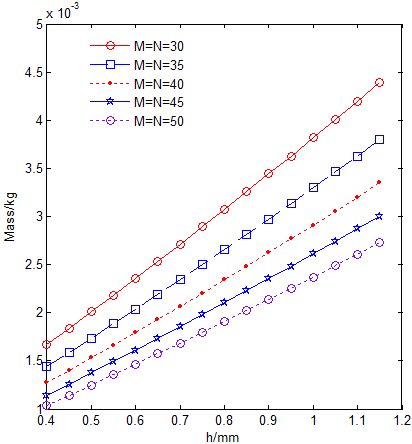


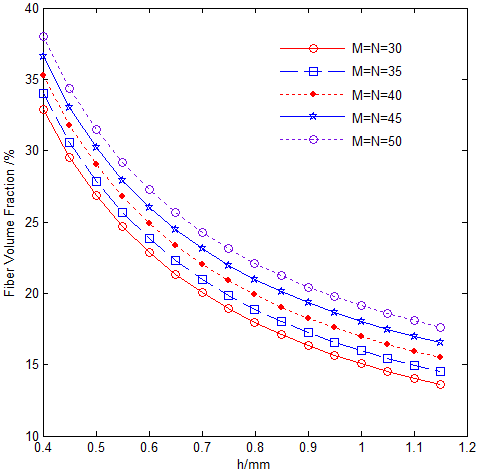

Supplement: S2 File — (DOCX) [file pone.0254691.s002.docx]
