# Supplementary material for: Parametric characteristics analysis of three cells in 3D and five-directional annular braided composites
Source: PLoS One. 2021 Aug 4;16(8):e0254691. doi: 10.1371/journal.pone.0254691 (PMC8336883; doi:10.1371/journal.pone.0254691)

**%%%Fig17 program source code**

clc;clear all;

S_1=0.031;S_2=0.0081;w_h=0.4;

h=0.4:0.05:1.2;R_in=5;R_out=R_in+h;M=50;N=0;

rou_1=1.8e-3;%%% 碳纤维密度，单位：g/mm3

rou_2=0.9e-3;%%% 基体密度，单位：g/mm3

for i=1:(length(h)-1)

A_1(i)=sqrt(((R_in+0.75.*h(i))*sin(pi/(M+N))).^2+(w_h/2).^2+(((R_in+0.75*h(i))*cos(pi/(M+N)))-R_in-0.5.*h(i)).^2);

aerfa_1(i)=acos(0.25*h(i)/A_1(i));

aerfa_11(i)=360/(2*pi)*acos(0.25*h(i)/A_1(i));

A_2(i)=sqrt(((R_in+0.25*h(i))*sin(pi/(M+N))).^2+(w_h/2).^2+(((R_in+0.25*h(i))*cos(pi/(M+N)))-R_in).^2);

aerfa_2(i)=acos(0.25*h(i)/A_2(i));%%%弧度

aerfa_22(i)=360/(2*pi)*acos(0.25*h(i)/A_2(i));

A_3(i)=sqrt(((R_in+1.*h(i))*sin(2*pi/(M+N))-(R_in+0.75.*h(i))*sin(pi/(M+N))).^2+(w_h/2).^2+((R_in+1.*h(i))*cos(2*pi/(M+N))-(R_in+0.75.*h(i))*cos(pi/(M+N))).^2);

aerfa_3(i)=acos(0.25*h(i)/A_3(i));%%%弧度

aerfa_33(i)=360/(2*pi)*acos(0.25*h(i)/A_3(i));

A_4(i)=sqrt((((R_in+0.25*h(i))*sin(pi/(M+N)))-(R_in+0.5*h(i))*sin(2*pi/(M+N)))^2+(w_h/2)^2+(((R_in+0.25*h(i))*cos(pi/(M+N)))-(R_in+0.5*h(i))*cos(2*pi/(M+N)))^2);

aerfa_4(i)=acos(0.25*h(i)/A_4(i));%%%弧度

aerfa_44(i)=360/(2*pi)*acos(0.25*h(i)/A_4(i));

A_top(i)=sqrt((((R_in+0.25*h(i))*sin(2*pi/(M+N)))-(R_in+0.75*h(i))*sin(pi/(M+N)))^2+(w_h/2)^2+(((R_in+0.25*h(i))*cos(2*pi/(M+N)))-(R_in+0.75*h(i))*cos(pi/(M+N)))^2);

aerfa_top(i)=acos(0.25*h(i)/A_top(i));%%%弧度

aerfa_topp(i)=360/(2*pi)*acos(0.25*h(i)/A_top(i));

A_low(i)=sqrt((((R_in+0.75*h(i))*sin(2*pi/(M+N)))-(R_in+0.25*h(i))*sin(pi/(M+N)))^2+(w_h/2)^2+(((R_in+0.75*h(i))*cos(2*pi/(M+N)))-(R_in+0.25*h(i))*cos(pi/(M+N)))^2);

aerfa_low(i)=acos(0.25*h(i)/A_low(i));%%%弧度

aerfa_loww(i)=360/(2*pi)*acos(0.25*h(i)/A_low(i));

end

plot(0.4:0.05:1.15,aerfa_11,'*')

hold on

plot(0.4:0.05:1.15,aerfa_22,'+')

hold on

plot(0.4:0.05:1.15,aerfa_33,'r')

hold on

plot(0.4:0.05:1.15,aerfa_44,'b')

hold on

plot(0.4:0.05:1.15,aerfa_topp,'y')

hold on

plot(0.4:0.05:1.15,aerfa_loww,'+r')

hold on

xlabel('h/mm');ylabel('Braided Angle \circ');


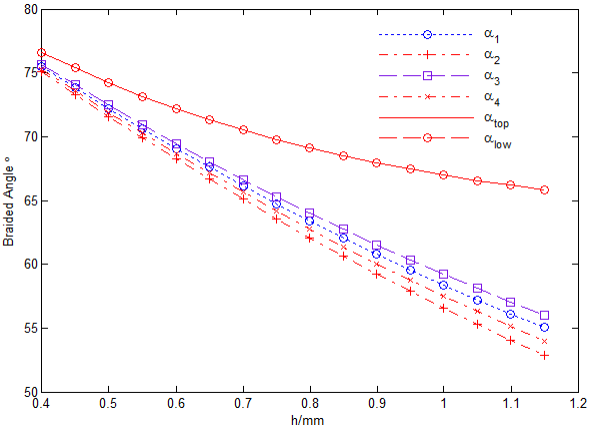

Supplement: S3 File — (DOCX) [file pone.0254691.s003.docx]
